# External Auditory Canal Metastasis of Colorectal Adenocarcinoma: A Comprehensive Case Report

**DOI:** 10.1002/ccr3.72569

**Published:** 2026-04-18

**Authors:** Luna Sissa, Stefano Cardelli, Deborah Malvi, Gilberto Poggioli, Matteo Rottoli, Giacomo Calini

**Affiliations:** ^1^ Surgery of the Alimentary Tract IRCCS Azienda Ospedaliero‐Universitaria di Bologna, Policlinico di S. Orsola Bologna Italy; ^2^ Department of Medical and Surgical Sciences Alma Mater Studiorum‐University of Bologna Bologna Italy; ^3^ Pathology Unit IRCCS Azienda Ospedaliero‐Universitaria di Bologna, Policlinico di S. Orsola Bologna Italy

**Keywords:** colorectal cancer, diagnostic imaging in oncology, external auditory canal, metastasis, rare metastatic sites

## Abstract

Metastatic colorectal adenocarcinoma with a single‐site metastasis to the external auditory canal is an exceedingly rare phenomenon. A multidisciplinary approach involving collaboration among various medical specialties is essential for optimizing patient care and improving survival outcomes.

**Abbreviations**
CAPOX: Capecitabine plus oxaliplatin
CN: Cranial nerve
CRC: Colorectal cancer
CT: Computed tomography
EAC: External auditory canal
EGFR: Endothelial growth factor receptor
FDG PET: Fludeoxyglucose‐18 positron emission tomography
FOLFOX: Leucovorin, fluorouracil, and oxaliplatin
MMR: Mismatch repair
MRI: Magnetic resonance imaging
VEGF: Vascular endothelium growth factor

## 1 Introduction

Metastasis to the external auditory canal (EAC) is uncommon and generally indicative of advanced disease. While metastases from other primary sites, such as breast, lung, and renal carcinomas, have been documented, metastasis from intestinal adenocarcinoma to other sites is exceedingly rare. To our knowledge, there are only two reports of intestinal adenocarcinoma metastasis to the external ear canal in the literature [1, 2]. This comprehensive case report aims to provide insights into the diagnostic workup, treatment approach, and clinical outcomes of a patient with metastatic intestinal adenocarcinoma involving the EAC.

## 2 Case History

A 58‐year‐old woman presented to our attention with a history of new‐onset headaches and paralysis of the facial nerve (VII CN) responsive to steroid treatment. The patient had no relevant comorbidities. She underwent a quadrantectomy 20 years before for an infiltrating ductal carcinoma of the right breast, pT1 N0 M0. She also had a positive family history of throat (grandfather), breast (grandmother), and prostatic neoplasia (father).

## 3 Methods

### 3.1 Diagnostic Workup

**FIGURE 1:**
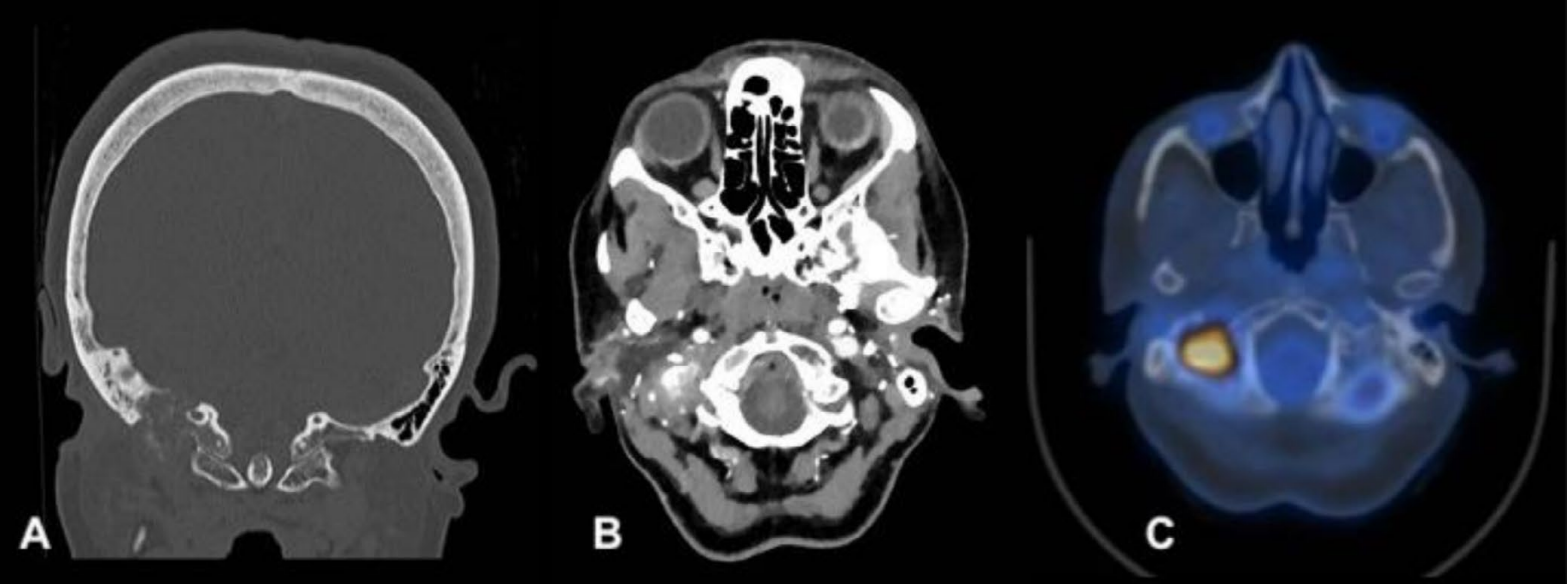
Expansive lesion at the external auditory canal (23 × 20 × 12 mm), localized to the right posterior foramen lacerum, with osteolysis of the rocca petrosa (A), indissociable from the internal jugular vein (B), and with an increased FDG uptake (SUV max 21.4) at the PET/TC scan (C).

**FIGURE 2:**
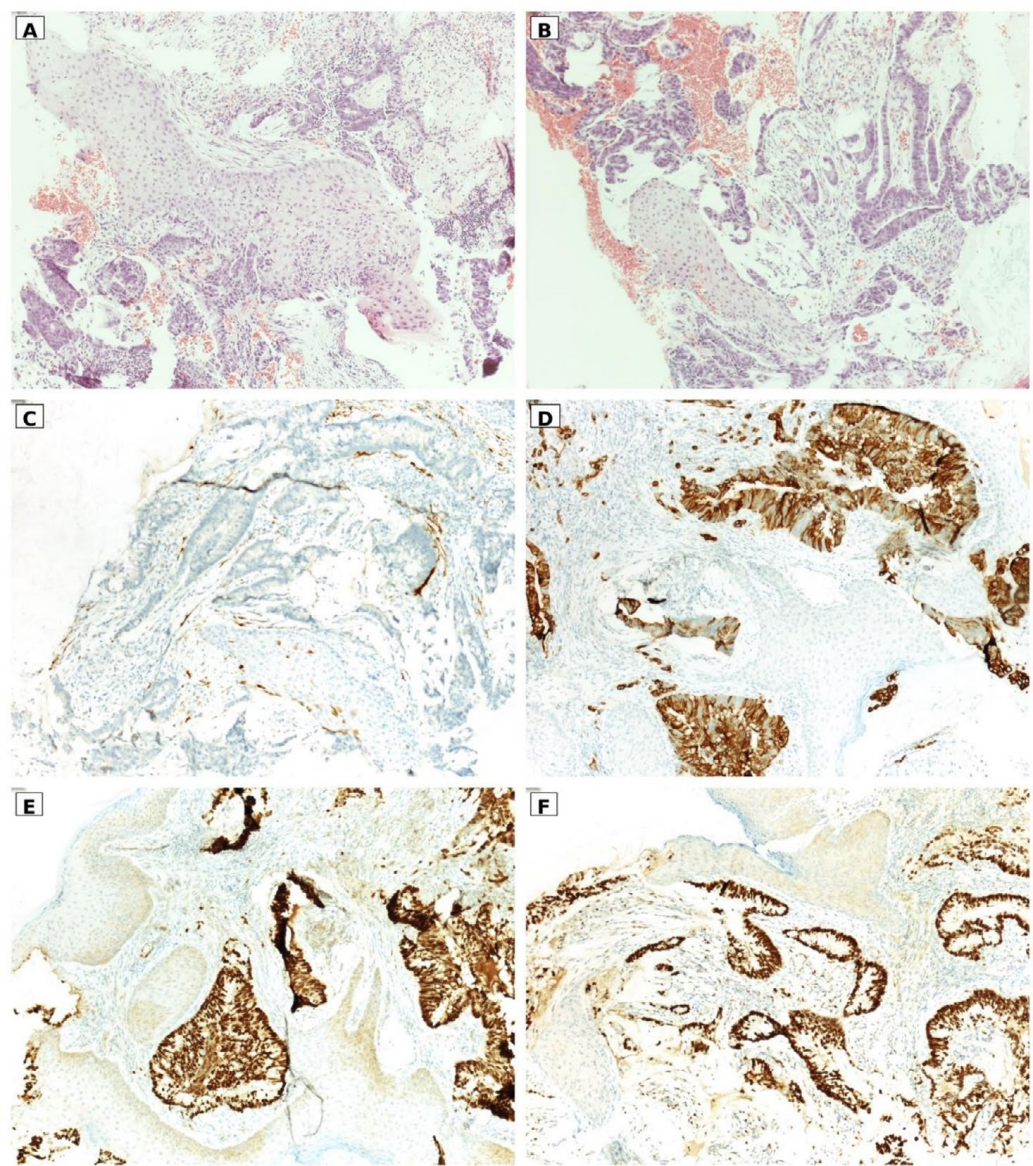
Panels A and B show histological sections of the lesion stained with hematoxylin and eosin (original magnification ×20). Panels C and D illustrate the keratin immune‐phenotype of the neoplasm, showing strong immunoreactivity for cytokeratin 20 (D, original magnification ×10) and complete negativity for cytokeratin 7 (C, original magnification ×10). The neoplasm also showed strong immunoreactivity for both CDX2 (E, original magnification ×10) and SATB2 (F, original magnification ×10).

In consideration of the new symptoms and positive personal and familial anamnesis of cancer, she underwent head MRI and CT scan, with evidence of an expansive lesion measuring 23 × 20 × 12 mm, localized to the right posterior foramen lacerum, with associated osteolysis of the ipsilateral rocca petrosa (Figure 1A). The lesion was poorly dissociable from the internal jugular vein (Figure 1B). Upon agobiopsy, the lesion was characterized as a mucin‐producing intestinal‐type poorly differentiated carcinoma. Specifically, the histological assessment of the biopsy specimen showed mucosa lined by squamous epithelium, consistent with EAC mucosa, infiltrated by an adenocarcinoma with focal mucin‐secreting features. The morphology of the neoplasm, composed of cells with pencil‐like nuclei and angulated glands, showed a keratin and immunohistochemical profile consistent with a tumor of gastrointestinal origin; in particular, the dual immunoreactivity for CDX2 and SATB2 supported a colonic origin of the neoplastic process (Figure 2).

**FIGURE 3:**
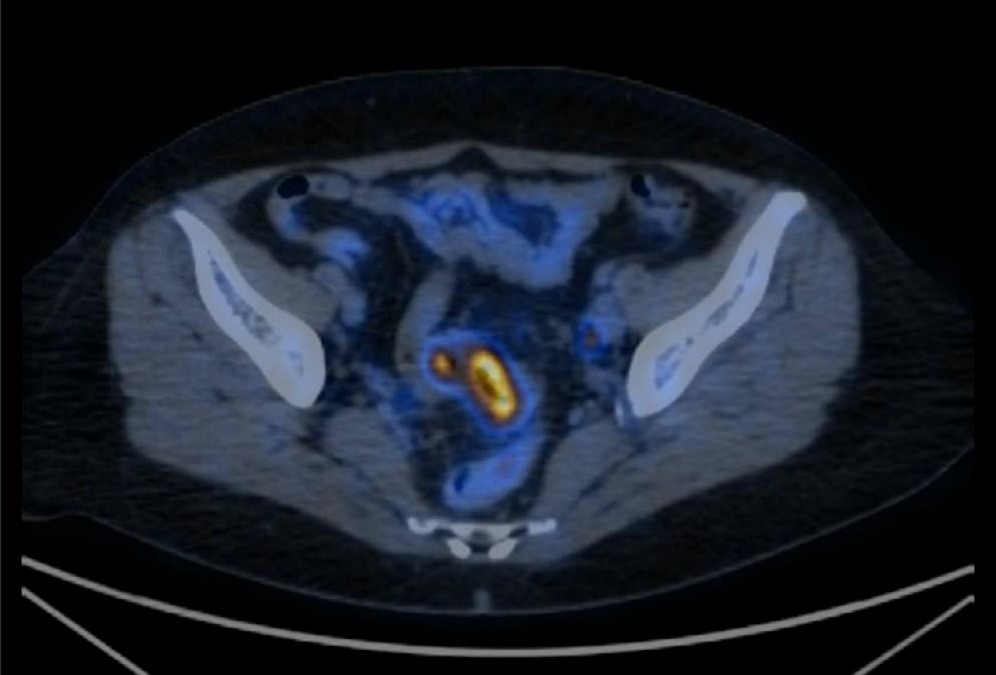
The FDG PET showing rectosigmoid junction colorectal cancer and local lymph node involvement. Clinical staging: CT3–4 N+ M1.

Considering the histological profile of the lesion, the patient underwent an esophagogastroduodenoscopy and colonoscopy, finding a circumferential stenotic lesion of the distal sigmoid colon at the rectosigmoid junction (which, upon biopsy, was characterized as an infiltrating colon adenocarcinoma, pMMR) and FDG PET with evidence of local lymph node involvement adjacent to the sigmoid lesion (Figures 1 and 3). Diagnostic workup confirmed the presence of a primitive adenocarcinoma in the distal sigmoid colon with locoregional lymphoid spread and a metastasis to the EAC (cT3‐4 N+ M1).

## 4 Conclusions and Results

**FIGURE 4:**
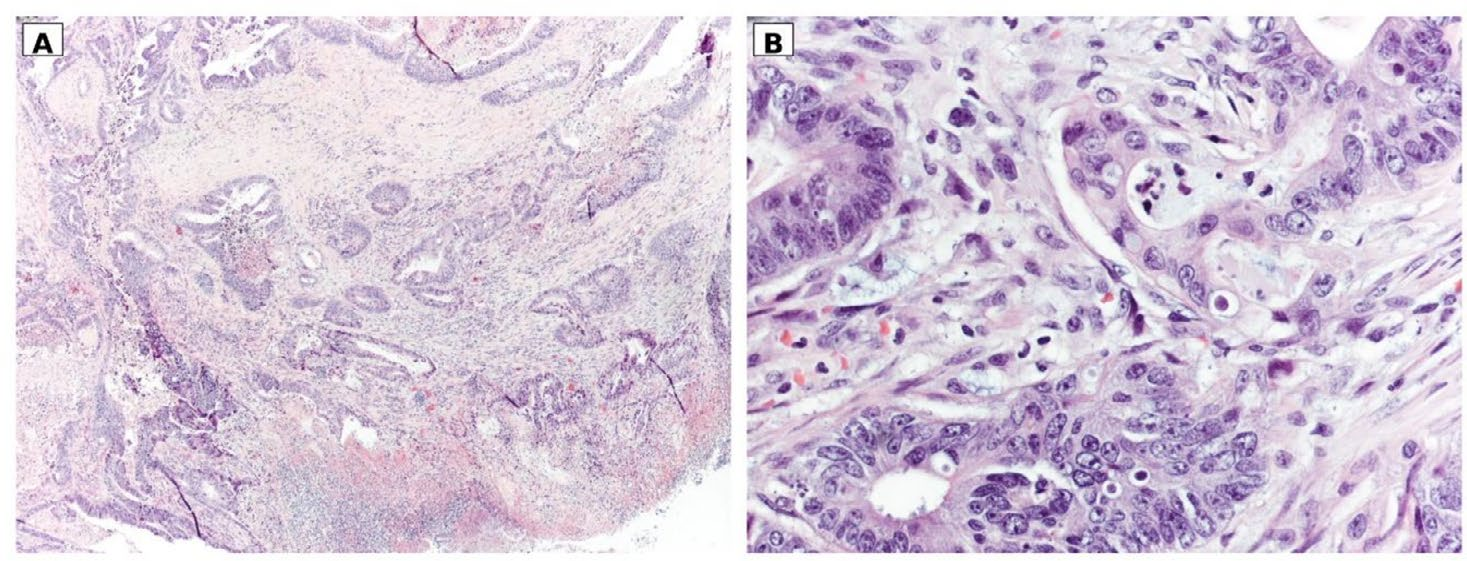
Panels A and B show histological sections of the colonic lesion resected by colectomy, stained with hematoxylin and eosin, at original magnifications of ×4 (A) and ×40 (B). The neoplasm shows full‐thickness infiltration of the bowel wall (A) and, at higher magnification, displays morphological features overlapping with those observed in the previous biopsy specimen (see figure 2).

The EAC metastasis was deemed not amenable to surgical excision in light of its extension and involvement of major vascular structures. The primitive lesion was treated surgically through robotic anterior rectal resection with partial mesorectal excision. The patient had an uneventful postoperative course and was discharged on postoperative day 5. The definitive histological evaluation profile of the lesion was intestinal adenocarcinoma of the rectosigmoid junction, low‐grade G2, pT3 N1b (2/13) with tumor deposits in the perivisceral adipose tissue (max 0.8 cm, N1c), M1 EAR, high‐grade budding, LVI+, PNI−, R0, and pMMR (Figure 4).

After surgical resection, she was referred to her local oncology center. Peripheral blood molecular analysis showed a polymorphism of the UGT1A1 gene (UGT1A1*28) associated with high toxicity of irinotecan‐like antiblastic drugs. The patient was started on chemotherapy with the FOLFOX scheme. Bevacizumab was contraindicated in light of the right jugular vein infiltration. She underwent 12 FOLFOX cycles followed by eight radiotherapy sessions. Radiotherapy targeted the EAC metastasis with a CyberKnife‐based stereotactic radiotherapy (total dose 32Gy at 80% isodose fractionated in eight sessions).

### 4.1 Follow‐up

At 2‐year follow‐up, no local recurrence or other metastasis was found. The head MRI demonstrated a reduction in the overall volume of the secondary mass, which, while still not dissociable from the internal jugular vein, appeared increasingly inhomogeneous and poorly enhanced. The patient reported an overall reduction in headache frequency and no recurrence of CN VII paralysis.

## 5 Discussion

Metastatic spread of colorectal cancer (CRC) to the EAC represents an unusual and challenging clinical scenario.

CRC is one of the most common malignancies worldwide, with a significant burden on global health. According to the World Cancer Research Fund, CRC ranks third in terms of incidence and second in terms of mortality. The incidence of CRC varies geographically, with higher rates in developed countries, and is influenced by lifestyle factors such as a Western diet, sedentary lifestyle, and aging. Familial predisposition, genetic mutations (e.g., APC, KRAS, and TP53), and inflammatory bowel diseases (e.g., Crohn's disease and ulcerative colitis) are established risk factors for CRC development [3, 4, 5, 6, 7].

The natural history of CRC typically involves local invasion, lymphatic spread, and metastases to the liver and lungs, which are the most common sites of distant metastases. However, metastasis to rare sites such as the EAC highlights the heterogeneity of CRC behavior and poses diagnostic and therapeutic challenges [4].

The presented case of a 58‐year‐old woman with metastatic intestinal adenocarcinoma involving the EAC emphasizes the importance of comprehensive diagnostic evaluation and multidisciplinary management. The patient's history of breast cancer and positive family history of neoplasia underlines the significance of thorough clinical assessment and surveillance in individuals with genetic predisposition or previous malignancies.

Diagnostic modalities, such as MRI, CT scans, and FDG PET, play crucial roles in identifying metastatic lesions and primary tumors, thereby guiding treatment decisions. Histological confirmation through biopsy is crucial for accurate diagnosis and determining tumor characteristics, including differentiation grade, molecular profile, and biomarker expression [7].

Therapeutic strategies for metastatic CRC encompass a multimodal approach, including surgery, chemotherapy, targeted therapy, and immunotherapy. Surgical resection of the primary tumor and metastatic lesions remains the cornerstone of curative‐intent treatment, particularly in cases amenable to complete resection. However, the extent of surgical intervention depends on factors such as tumor location, size, extent of metastasis, and the patient's overall health status [7].

Chemotherapy regimens such as CAPOX (capecitabine plus oxaliplatin) and FOLFOX (leucovorin, fluorouracil, and oxaliplatin) have demonstrated efficacy in prolonging survival and improving quality of life in metastatic CRC. Targeted therapies targeting specific molecular pathways, such as anti‐EGFR antibodies (cetuximab and panitumumab) and anti‐VEGF agents (bevacizumab), have revolutionized the management of metastatic CRC, particularly in patients with wild‐type RAS/BRAF tumors [7].

In the presented case, the decision to perform robotic anterior rectal resection with partial mesorectal excision for the primary lesion in the rectosigmoid junction reflects the importance of aggressive surgical management in selected patients with metastatic CRC. Adjuvant chemotherapy following surgical resection aims to eradicate micrometastasis and prevent disease recurrence, with the CAPOX or FOLFOX regimens being a standard‐of‐care option [7].

However, therapeutic decisions must be individualized based on tumor characteristics, patient preferences, and treatment goals. The infiltration of major vascular structures by the secondary mass in the EAC highlights the limitations of surgical resection and the need for alternative treatment modalities, such as systemic chemotherapy and local radiotherapy.

In the previously reported cases, the diagnosis of EAC metastasis was established histologically following excisional biopsy of the lesion. In both cases, the management was necessarily palliative [1, 2]. In Carson's report, complete excision of the EAC mass was performed under local anesthesia and led to a marked improvement in hearing [1]. Sequential chemotherapy regimes aimed at systemic disease control resulted in transient stabilization followed by multiorgan progression, and the patient died approximately 6 months after presentation. In Carr & Anderson's report, the excision is described as a diagnostic biopsy rather than a complete exeresis, followed by systemic chemotherapy upon evidence of disseminated disease [2]. The patient's general condition deteriorated despite treatment, with death occurring 5 months after diagnosis. In no case did the patient receive radiotherapy to the temporal bone. Oncological outcomes were poor in both reports, but significant in terms of symptomatic relief. The authors emphasized the importance of palliative local management to address quality of life, even when the oncologic prognosis is dismal [1, 2].

This case report describes improved symptomatic relief and a 2‐year survival following diagnosis, with no local recurrence or other systemic metastasis. Comprehensive diagnostic evaluation, multidisciplinary collaboration, and personalized treatment approaches are essential for optimizing patient oncologic outcomes and quality of life. In our case, the management of metastatic colorectal adenocarcinoma involving the EAC required a multidisciplinary approach involving surgical oncologists, medical oncologists, radiation oncologists, and otolaryngologists. Further research into novel therapeutic strategies and predictive biomarkers will help to improve prognosis and quality of life in patients with metastatic CRC. As in the previously reported cases, the management consists of the surgical resection of the primary tumor combined with chemotherapy and radiotherapy.

In conclusion, metastatic spread of intestinal adenocarcinoma to the EAC is an exceedingly rare phenomenon. A multidisciplinary approach involving collaboration among various medical specialties is essential for optimizing patient care and improving survival outcomes.

## Author Contributions

**Luna Sissa:** data curation, investigation, project administration, writing – original draft. **Stefano Cardelli:** investigation, writing – review and editing. **Deborah Malvi:** investigation, writing – review and editing. **Gilberto Poggioli:** investigation, resources, supervision, writing – review and editing. **Matteo Rottoli:** conceptualization, investigation, resources, supervision, writing – review and editing. **Giacomo Calini:** data curation, investigation, methodology, project administration, resources, supervision, writing – original draft.

## Funding

This work was supported by Università di Bologna, OA fee by the national CARED‐CRUI agreement.

## Consent

Written informed consent was obtained from the patient to publish this report per the journal's patient consent policy.

## Conflicts of Interest

The authors declare no conflicts of interest.

## Data Availability

The data supporting this study's findings are available in the article.
